# Immunophenotypic characterization of human T cells after *in vitro* exposure to different silicone breast implant surfaces

**DOI:** 10.1371/journal.pone.0192108

**Published:** 2018-02-08

**Authors:** Giuseppe Cappellano, Christian Ploner, Susanne Lobenwein, Sieghart Sopper, Paul Hoertnagl, Christina Mayerl, Nikolaus Wick, Gerhard Pierer, Georg Wick, Dolores Wolfram

**Affiliations:** 1 Department of Plastic, Reconstructive and Aesthetic Surgery, Medical University of Innsbruck, Innsbruck, Austria; 2 Department of Internal Medicine V, Medical University of Innsbruck, Innsbruck, Austria; 3 Central Institute for Blood Transfusion & Immunological Department, Medical University of Innsbruck, Innsbruck, Austria; 4 Department of Internal Medicine III, Cardiology and Angiology, Medical University of Innsbruck, Innsbruck, Austria; 5 Wick Laboratory, Innsbruck, Austria; 6 Division of Experimental Pathophysiology and Immunology, Biocenter, Medical University of Innsbruck, Innsbruck, Austria; Universita degli Studi di Roma La Sapienza, ITALY

## Abstract

The most common complication of silicone breast implants is capsular contracture (massive scar formation around the implant). We postulate that capsular contracture is always a sequel to inflammatory processes, with both innate and adaptive immune mechanisms participating. In general, fibroblasts and macrophages have been used as cell types to evaluate *in vitro* the biocompatibility of breast implant surfaces. Moreover, also T cells have been found at the implant site at the initial stage of fibrous capsule formation. However, only few studies have addressed the influence of surfaces with different textures on T-cell responses. The aim of the present study was to investigate the immune response of human peripheral blood mononuclear cells (PBMC) to commercially available silicone breast implants in *vitro*. PBMC from healthy female blood donors were cultured on each silicone surface for 4 days. Proliferation and phenotype of cultured cells were assessed by flow cytometry. Cytokine levels were determined by multiplex and real-time assay. We found that silicone surfaces do not induce T-cell proliferation, nor do they extensively alter the proportion of T cell subsets (CD4, CD8, naïve, effector memory). Interestingly, cytokine profiling identified matrix specific differences, especially for IL-6 and TNF-α on certain surface topographies that could lead to increased fibrosis.

## Introduction

Breast augmentation with silicone mammary implants (SMI) is one of the most commonly performed procedures in aesthetic surgery [1]. According to the American Society of Plastic Surgeons (ASPS), officially more than 200.000 breast augmentations were performed in USA alone in 2015 [2]. Interestingly, the International Society of Plastic and Regenerative Surgeons (ISPRES) reported that in 2013, 1.7 million breast augmentations were performed in the rest of the world, notably in Latin America and Europe [3]. The incidence of local complications in SMI carriers, though frequently reported is controversial [4]. The most common complication of SMI is capsular contracture, with a combined overall rate of 10.6% [5]. The variability in reported capsular contracture rates depends on many factors such as different time spans, types of implant used, implant locations, and others termed “defensive reporting”. We postulate that capsular contracture is always a sequela of inflammatory processes, in which both innate and adaptive immune mechanisms take part [6]. We also suggest that silicone degradation products in the body might activate cells from both, the innate and the adaptive immune system, and thus might perpetuate a chronic pro-inflammatory response in the local tissue [7].

The chronic inflammatory stimulus might also be an important cause for the development of anaplastic large cell lymphoma (ALCL), which is a rare T-cell lymphoma developing in the capsule surrounding the breast implants [8]. Though its etiology is unknown, aside chronic inflammation, bacterial infections (biofilm) and tumor immunological escape are discussed as mechanisms in the development of breast implant-associated anaplastic large cell lymphoma (BIA-ALCL) [9].

In our previous study, we analyzed the cellular and molecular composition of fibrous capsules removed from patients at various times after surgery [10]. Immunohistochemical analysis showed the presence of macrophages, dendritic cells (DC), fibroblasts and mainly activated CD4+ T-cells expressing CD25 and CD45RO markers at the capsule/silicone implant contact zone [10]. Flow cytometry analysis (FACS) showed a preponderance of effector T cells (Teffs) (mainly TH17 cells) within the peri-SMI fibrotic capsule, and a decreased number of regulatory T cells (Tregs), which correlated with increasing severity of capsular fibrosis, while maintaining their suppressive activity [11].

Meta-analyses showed that capsular contracture is more frequent with smooth implants [12,13]. However, much effort has been invested in investigating and improving the surface topographies with the aim to prevent capsular contracture [14]. In particular, it was shown that both -the smooth and textured implant surfaces- have micro- and nanoscale topographies that can influence wound healing and the immune response [15]. Recently, Barr et al. introduced a new classification of implant texture according to implant roughness. This new classification now identifies 4 different implant types, namely macro-, micro-, meso-, and nano-textured once [16].

Microtextured and nanotextured surfaces may influence the attachment, proliferation, migration and differentiation of several cell types [17,18].

Traditionally, fibroblasts and macrophages have been used as cell types to evaluate the biocompatibility of breast implant surfaces [19]. However, lymphocytes (mainly T cells) have also been found at the implant site at the initial stage of fibrous capsule formation [20]. It has been suggested that the continued and prolonged stimulation of lymphocytes could lead to a clonal expansion of an activated T-cell pool, which in time, will acquire mutations allowing malignant transformation of these cells into BIA-ALCL [21–23].

Thus it would appear that lymphocytes also play a key role as cellular determinants in the outcome of biocompatible breast implants. However, only few studies have addressed *in vitro* the direct effects of silicone surfaces on peripheral blood mononuclear cells (PBMC). PBMC comprise mainly of lymphocytes (T, B and NK cells), monocytes, and dendritic cells (DC). In humans, the frequencies of these populations might vary across individuals, with lymphocytes typically representing 80% of PBMCs, monocytes in the range of 10–30% of PBMCs, and DC being rare (1–2%).

Meza Britez et al. found increased numbers of CD3+ T cells and also of cellular infiltrates (macrophages) in capsular biopsies recovered from textured rather than smooth implants [20]. Based on their findings, the authors concluded that textured silicone might induce a local T-cell response [20]. Another study in which nano- and microsilicone particles were used to investigate indirectly the effect of breast silicone surfaces on T-cell response [24] reported that nanosilicone reduced the immune responses in the sense that there was reduced secretion of the proinflammatory cytokines IL-6, TNF-α and IFN-ᵧ in comparison to silicone microparticles used at the same concentration [24].

The present study was aimed at investigating the *in vitro* immune response of human PBMC to silicone implants to which the body tissue will be exposed *in vivo*.

## Material and methods

### Ethics statement

This study was approved by the Ethics Committee of the Medical University of Innsbruck. Written consent was obtained from all donors (approval number: AN2016-0108 362/4.19).

### Blood specimen collection

Peripheral blood was obtained from seven healthy female donors (20–50 years old) in cooperation with the Central Institute for Blood Transfusion & Immunological Department, Tirol Clinic Ltd., Innsbruck, Austria. Exclusion criteria of this study were as follows: history of breast implant reconstruction, autoimmune or rheumatic diseases, history of kidney failure, occurrence of HIV or hepatitis infections, diabetes, pregnancy, anti-inflammatory or immunosuppressant therapy, presence of other silicone devices implanted in the body (i.e. gastric band, ear implants, etc.). In every single female donor all 7 surfaces were investigated for their biocompatibility.

### Silicone surfaces preparation

Circular samples of 16 mm were cut from each sterile silicone implant shell under sterile conditions.

Seven implant shells were tested in this study:

a) SilkSurface® (Establishment Labs Coyol Free Zone, Alajuela, Costa Rica)
b) VelvetSurface® (Establishment Labs Coyol Free Zone, Alajuela, Costa Rica)
c) Allergan Smooth surface (Allergan Medical Corporation, Santa Barbara, Calif)
d) Allergan Biocell surface (Allergan Medical Corporation, Santa Barbara, Calif)
e) Polytech Texture surface (Polytech. Dieberg, Germany)
f) Polytech Micropolyurethane foam (Polytech, Dieberg, Germany)
g) Mentor Siltex surface (Mentor Corporation, Santa Barbara Calif)

Representative images of each surface were taken using Nikon SMZ800N stereomicroscope (Carl Zeiss, Austria) linked to a ProGres CT3 camera controlled by ProGres capture pro software (version 2.9.0.1). Scanning electron microscope (SEM) analysis of each surface or surfaces used in this study has already been shown in [16, 25].

### Cell isolation and *in vitro* culture

PBMC were isolated from whole blood using Lymphoprep (Axis-Shield, Oslo, Norway). PBMC from each donor were seeded on each surface and cultured in 24-well plates (Sarsted, Nümbrecht, Germany) for 4 days in RPMI 1640 medium (Lonza, Walkersville, MD) supplemented with 10% fetal bovine serum (FBS) (GE Healthcare, Piscataway, NJ), 2 mM glutamine and 100 U/ml of penicillin and streptomycin (Lonza) at 37°C in a 5% CO_2_. As control, cells were seeded directly onto tissue culture polystyrene well plate (Sarstedt).

### Flow cytometry analysis

On day 4 cells were harvested from cultures. Viable cells were counted by trypan blue exclusion (Sigma-Aldrich, St.Louis, MO). Then cells were washed and stained with monoclonal antibodies against the following surface markers: CD3-Alexa Fluor®700 (clone:SP34-2) (BD Pharmigen™, Franklin Lakes, NJ), CD4-PerCP-Cy™ 5.5 (L200) (BD Pharmigen™), CD8-APC-Cy™7 (SK1) (BD Pharmigen™), CD25-PE/Cy7 (M-A251) (Biolegend, San diego, CA), CD197-PE (G043H7) (Biolegend) and CD45RA-ECD (J.33) (Beckman Coulter, Brea, CA). To discriminate between live and dead cells, cells were stained with the Fixable Viability Dye eFluor® 520 (eBioscience, San Diego, CA). For intracellular anti-Foxp3 staining, permeabilized cells were stained with anti-Foxp3-APC (236A/7) according the manufacturer's instructions (eBioscience). The clone 236A/7 was used, as previously described [26]. Finally, cells were acquired on a BD LSRFortessa™ flow cytometer using FACSDIVA software 6.1.3 (BD Biosciences). Eight parameter analyses were performed by using FlowJo 9.6 software (FlowJo, Ashland, OR, USA). Gating strategies are shown in the Results section.

### Proliferation assay

On day 0, PBMC from each donor were labeled with carboxyfluorescein diacetate succinimidyl ester (CFSE) (Molecular Probes, Eugene, Oregon) and seeded on each surface in the presence or absence of human anti-CD3 (1 μg/ml, clone OKT3) and anti-CD28 (3 μg/ml, clone CD28.2) (eBioscience) monoclonal antibodies (mAbs) that were used as polyclonal stimuli. On day 4, proliferation of CFSE-labeled cells was assessed by flow cytometry.

### RNA isolation, cDNA synthesis and quantitative real-time polymerase chain reaction (PCR)

After 4 days of culture, supernatant was collected for later analyses. Adherent cells were detached using Trypsin (Sigma) for 5 min at 37° C. Detached cells were transferred into a new tube and washed one time with PBS. RNA was isolated by adding 500 μl of Trizol reagent (MRC Inc. Cincinnati, OH, USA), and cDNA was synthesized using random hexamer primers and iScript cDNA-synthesis kit (Biorad, Germany). The PCR reactions were performed using the SsoAdvanced™ Universal SYBR® Green Supermix kit (Biorad, Germany) in a CFX96 (Biorad, Germany) using the following protocol: 95°C for 3 min, 40 cycles of 95°C (15s), 60°C (15s), and 72°C (10s). Gene expression was determined by using the Bio-Rad CFX Manager 3.1 software, and CT values were normalized to the mean of the reference gene ribosomal 18S rRNA. All primers used in this study (Table 1) were synthesized by Microsynth (Austria) and specificity of PCR products was confirmed by analysis of the melting curve.

**Table 1:** 

Gene	Sense primer	Antisense primer
CD14	5‘-AGCCAAGGCAGTTTGAGTCC-3‘	5‘-TAAAGGACTGCCAGCCAAGC-3‘
CD16	5‘-CACCATCACTCAAGGTTTGG-3‘	5‘-AGTCCTGTGTCCACTGCAAA-3‘
CD68	5‘-GCTACATGGCGGTGGAGTACAA-3‘	5‘-ATGATGAGAGGCAGCAAGATGG-3‘
IL-1β	5'-ACAGATGAAGTGCTCCTTCCA-3'	5'-GTCGGAGATTCGTAGCTGGAT-3'
IL-8	5'-ATGACTTCCAAGCTGGCCGTGGCT-3'	5'-TCTCAGCCCTCTTCAAAAACTTCTC-3'
IL-10	5'-AGGGAAGAAATCGATGACAGC-3'	5'-TCAAGGCGCATGTGAACTC-3'
TNF-α	5'-TTGAGGGTTTGCTACAACATGGG-3'	5'-GCTGCACTTTGGAGTGATCG-3'
TGF-β1	5'-CCCAGCATCTGCAAAGCTC-3'	5'-GTCAATGTACAGCTGCCGCA-3'
MCP-1	5'-GTCTTGAAGATCACAGCTTCTTTG-3'	5'-AGCCAGATGCAATCAATGCC-3'
18S rRNA	5'-GCAATTATTCCCCATGAACG-3'	5'-GGCCTCACTAAACCATCCAA-3'

### Multiplex assay

The cell culture supernatants were collected after 4 days and cytokine levels were quantified using ProcartaPlex®Multiplex Immunoassay according to manufacturer’s instructions (Affimetrix, eBioscience). This assay enables the detection of the following cytokines: GM-CSF, IFN-ᵧ, TNF-α, IL-10, IL-12p70, IL-13, IL-17A, IL-18, IL-1β, IL-2; IL-21; IL-22; IL-23; IL-27; IL-4; IL-5; IL-6; IL-9. The plate was run on a Luminex® MAGPIX instrument using xPonent 4.2 software (eBioscience).

### Data analysis

Statistical analysis was performed with GraphPad Prism5 (La Jolla, CA) using Friedman ANOVA test to compare the means. Dunn’s multiple comparison test was used as post-hoc test. The data are shown as mean± SEM. Differences were considered to be significant at *p< 0*.*05*.

## Results

### Proliferation and subsets of human T cells are not affected after exposure to silicone breast implant surfaces

First, we examined T-cell activation in PBMC cultured for 4 days on seven implant surfaces as shown in **S1 Fig**. Tissue culture polystyrene (plastic of the used microtiter plates) was included in this study as control material surface. In this study we assessed T-cell activation in response to different surfaces by analyzing the expression of CD25 marker. We found that CD25 expression did not exceed levels prior to plating **(S1 Table)**.

Next, we assessed T-cell proliferation as an additional/functional parameter to investigate T-cell activation. CFSE-labeled mononuclear cells were cultured on different silicone surfaces for 4 days and analyzed by FACS. There was no increased lymphocyte proliferation as shown by comparison to anti-CD3 and anti-CD28 polyclonal stimulation **(Panel A in S2 Fig).** Moreover, no differences were seen between different surfaces **(Panel B in S2 Fig)**. These data suggest that T cells do not proliferate in response to silicone breast implant surfaces alone.

**Fig 1:**
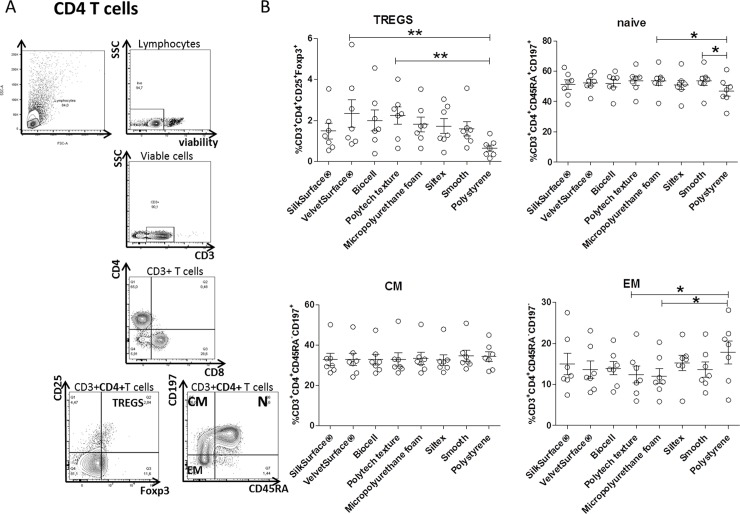
Immunophenotype of CD4+T cells cultured on silicone surfaces. A) Representative FACS plots showing gating strategy of 4 different CD4+ T cells. Briefly, a viability dye was used to discriminate live and dead cells. After gating on CD3+ T cells, Tregs and naïve /memory cells were discriminated on the basis of expression of CD25 and Foxp3 (Tregs) and CD45RA and CD197 (naïve/memory) markers, respectively. B) PBMC from 7 donors were cultured on different surfaces for 4 days. Cells were gated on CD4+ cells: Tregs (CD25^+^Foxp3^+^) naïve (N, CD45RA^+^CD197^+^), central memory (CM, CD45RA^-^CD197^+^), effector memory (EM, CD45RA^-^CD197^-^) were determined and shown as % of CD4+. Three independent experiments (2–3 donors/experiment) were performed. Results are expressed as mean ± SEM. Each data point represents an individual donor. Friedman ANOVA test was used to compare the means (Dunn’s multiple comparison test; *p<0.05, **p<0.01).

Previous *ex vivo* studies showed that the T-cell subpopulations that mainly react to silicone are CD4+ T cells [16, 27]. Therefore, we investigated the effect of surface structure on several T-cell subsets *in vitro* **(Fig 1A and 1B).** Cell viability was not affected by each surface on its own. FACS analysis showed on the majority of surfaces no altered proportion of naïve (N, CD45RA^+^CD197^+^), central memory (CM, CD45RA^-^CD197^+^) and effector memory (EM, CD45RA^-^CD197^-^), among CD4+ T cells. On the contrary, we observed a slight increase in the proportion of naïve T cells only in Micropolyurethane foam and smooth surfaces. Both Polytech surfaces induced a decrease in the proportion of effector memory cells in comparison to polystyrene **(Fig 1B).** We also observed a similar trend in the proportion of regulatory T cells (CD25^+^Foxp3^+^) in all surfaces in comparison to the smooth ones. However, an increase of Foxp3 expression was observed in PBMC cultured on VelvetSurface® and Polytech texture, in comparison to polystyrene.

**Fig 2:**
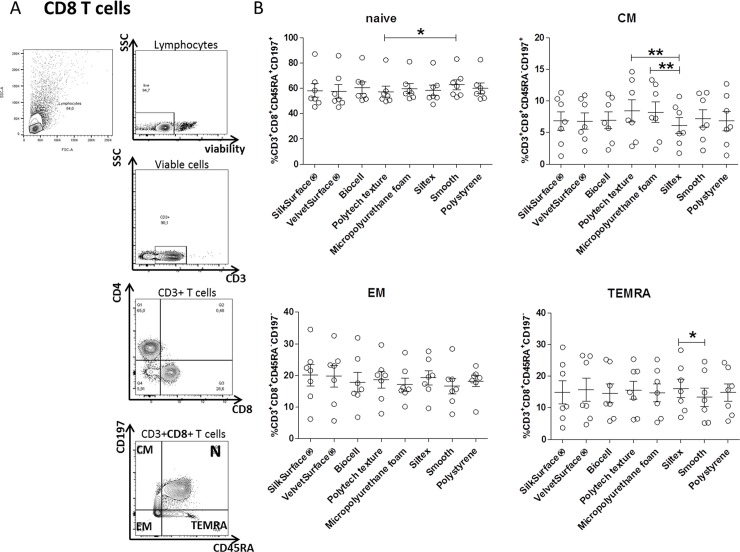
Immunophenotype of CD8+T cells cultured on silicone surfaces. A) Representative FACS plots showing gating strategy of 4 different CD8+ T cells. A viability dye was used to discriminate live and dead cells. After gating on CD3+ T cells, naïve /memory cells were discriminated on the basis of expression of CD45RA and CD197 markers, respectively. B) PBMC from seven donors were cultured on implants with different surfaces for 4 days. Cells were gated on CD8+ cells: naïve (N, CD45RA^+^CD197^+^), central memory (CM, CD45RA^-^CD197^+^), effector memory (EM, CD45RA^-^CD197^-^) and terminally differentiated central memory cells (TEMRA, CD45RA^+^CD197^-^) were determined and shown as % of CD8+. Three independent experiments (2–3 donors/experiment) were performed. Each data point represents an individual donor. Results are expressed as mean ± SEM. Friedman's ANOVA test was used to compare the means (Dunn’s multiple comparison test; * p<0.05,** p<0.01).

Similar to CD4+ T cell findings, FACS analysis of CD8+T cell subsets also revealed no changes in the proportion of naïve (N, CD45RA^+^CD197^+^), central memory (CM, CD45RA^-^CD197^+^), effector memory (EM, CD45RA^-^CD197^-^) and terminally differentiated central memory cells (TEMRA, CD45RA^+^CD197^-^). However, a minor increase in the proportion of naïve cells has been found on Polytech texture in comparison to the smooth surface and an increase in the proportion of CM cells has been found on both Polytech surfaces, in comparison to Siltex, which also showed a slight increased proportion of TEMRA in comparison to the smooth surface **(Fig 2B)**.

These data show that after exposure to silicone surfaces, the T-cell subset distribution is not extensively changed.

### Cytokine profile in response to silicone breast implant surfaces

As we did not detect substantial differences in individual T-cell subsets or proliferation, we aimed to determine T-cell paracrine activity. Therefore, we assessed the cytokine profile of PBMC response to different silicone surfaces. Supernatants collected on day 4 were analyzed by multiplex assay. This assay included macrophage activation cytokines (IL-1β, IL-6, IL-18 TNF-α, GM-CSF), cytokines important for macrophage fusion (IL-4, IL-13), anti-inflammatory cytokines (IL-10, IL-27), T cell-activation cytokines (IL-2 and INF-ᵧTH17 cytokines (IL-17, IL-21, IL-22, IL-23), TH9 cytokine (IL-9) and TH2 cytokine (IL-4). We did not detect IL-2 and IFN-ᵧ cytokines, which suggests that there was no T cell activation in response to the nature of silicone surface, confirming our previous findings by FACS analysis.

**Fig 3:**
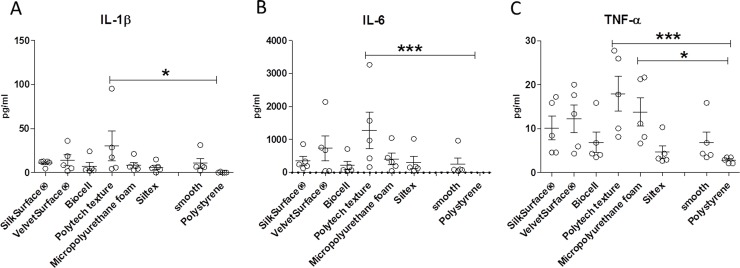
**IL-1β (A) IL-6 (B), and TNF-α(C) levels in the supernatants collected from PBMC cultured on 8 surfaces as indicated.** Results are expressed as mean ± SEM (n = 5). Each data point represents an individual donor. Friedman ANOVA test was used to compare the means (Dunn’s multiple comparison test; *p<0.05, ***p<0.001).

Among the other cytokines tested, only IL-1β, IL-6 and TNF-α were quantified **(Fig 3)**. The levels of the others were below the lower limit of quantification (LLOQ). Levels of IL-2 were close or below the LLOQ and thus not suitable for statistical comparison. PBMC cultured on polystyrene secreted neither IL-6 nor IL-1β cytokines but those on Polytech Texture surface showed increased levels of these two cytokines. Comparable IL-1β and IL-6 levels were found among all other surfaces tested **(Fig 3A and 3B).** Moreover, we also found increased TNF-αsecretion by PBMC cultured on Polytech Texture surface in comparison to polystyrene surface. Finally, TNF-α levels were also significantly increased in Polytech Micropolyurethane foam surface in comparison to polystyrene **(Fig 3C).**

### Influence of silicone breast implant surfaces on adherent cells

To investigate the contribution of those cells that directly counteract with the implant surfaces, we analyzed *in vitro* monocyte/macrophage markers and related cytokine expression of adherent cells using semi-quantitative RT-PCR. RNA was isolated from adherent cells cultured on different surfaces for 4 days.

**Fig 4:**
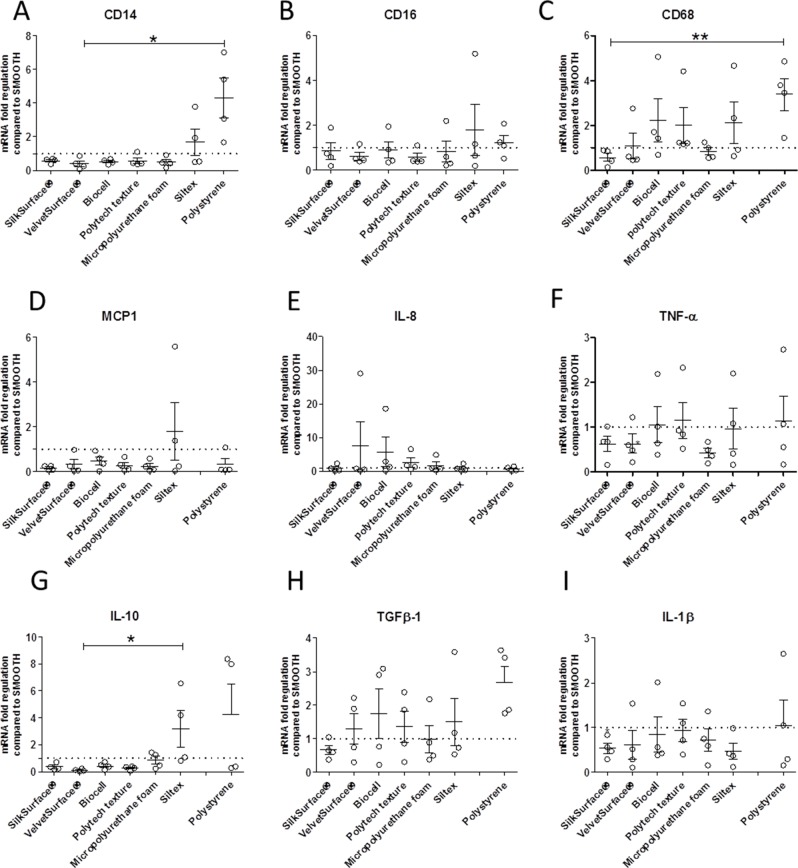
Quantitative RT-PCR analysis of CD14, CD16, CD68, IL-1 β, IL-8, TNF-α, TGF-β 1, IL-10 and MCP1 in adherent cells performed after 4 days of culture. Data are shown as mean ± SEM (n = 4) normalized to Smooth surface (dotted line). Each data point represents an individual donor. Friedman ANOVA test was used to compare the means (Dunn´s multiple comparison test; *p<0.05, **p<0.001).

Data were normalized for smooth surface to assess the influence of each specific texture on adherent cells. We found that CD14 was downregulated in all surfaces, except Siltex and polystyrene. Moreover, its expression on polystyrene and Siltex surfaces was increased in comparison to the other surfaces but this increase reached statistical significance only in comparison to VelvetSurface® **(Fig 4A).** CD14 is expressed by monocytes and macrophages, while macrophages express low CD14 and high CD16. CD68 mRNA (macrophages) was upregulated on Biocell, Polytech texture, Siltex and polystyrene surfaces and it was significantly decreased on SilkSurface® in comparison to polystyrene **(Fig 4C).** No differences were found in CD16, MCP-1, IL-8 mRNA expressions among the surfaces tested **(Fig 4B, 4D and 4E).**

Moreover, we found that TNF-α and IL-1β were slightly upregulated on Biocell (TNF-α, IL-1β), Polytech texture (TNF-α), Siltex (TNF-α), Polystyrene (TNF-α, IL-1β) in comparison to SilkSurface®, VelvetSurface® and Micropolyurethane foam surface (TNF-α) **(Fig 4F and 4I).** Regarding the anti-inflammatory cytokines, we also observed that IL-10 was downregulated on all surfaces except Siltex and polystyrene; the increase in Siltex reached statistical significance in comparison to VelvetSurface® (**Fig 4G**). Finally, TGF-β1 mRNA was upregulated in all surfaces, except on SilkSurface®, **(Fig 4H).**

## Discussion

Following SMI implantation, a natural foreign body reaction occurs with infiltration of macrophages and T cells into the site [27–28]. Our group has previously described the function of these lymphocytes by showing that they are mainly CD4+T cells with a profibrotic cytokine profile that mediates TH1/TH17 responses [11].

Therefore in the present study, we compared the in *vitro* T-cell response to six textured implants with smooth or polystyrene surfaces. Physical characterization (SEM, roughness, wettability) of those surfaces are described elsewhere [16, 18, 25]. Surface texture facilitates fibroblast ingrowth into interstices on textured surfaces, which would favor a thinner capsule without contraction compared to the smooth surface [13, 29].

In the first set of experiments, we investigated the effect of each surface on T- cell proliferation.

In general biomaterials are not considered immunogenic. However, it has been shown that biomaterials can act as mitogens and activate T cells since the functional group of the surface could interact with plasma glycoprotein on lymphocyte membrane [30]. CD69 is a typical activation marker of T cells but it is only detectable within 48 h [31]. In contrast, CD25 expression—which is the alpha chain of the IL-2 receptor—is more stable and persists for several days *in vitro* [32]. In our experimental settings, we cultured PBMC for 4 days and did not find significant differences in CD25 expression compared to the levels prior to plating. Furthermore, we found that none of the surfaces induced proliferation of T cells per se, nor did they significantly affect proliferation of CFSE-labeled PBMC when stimulated with anti- CD3/CD28 mAbs. Moreover, levels of IL-2 nor IFN-ᵧ cytokines were close or below the LLOQ in the supernatant of cultured PBMC. Our data are in agreement with the findings of Rodriguez et al. [33]. In their study, they tested different biomaterials (including silicone) and found that they did not activate T cells. In contrast, *ex vivo* analyses of fibrous capsules by others and our group showed the presence of activated T cells (with high expression of CD45RO and CD25 markers) at the implant site [10,20]. For the above-mentioned reasons, we cannot exclude that *in vivo*, the microenvironment might play a role in T-cell activation in response to silicone surface. Indeed, *in vitro* exposure to silicone surfaces did not change the subset of T cells, as we did not find for the majority of the surfaces differences in the ratio of CD4+ and CD8+ T cell subsets (naive, effector and memory) upon culture on the silicone surfaces. Interestingly, a slight increase in the proportion of naïve and memory/effector cells was found in Polytech texture surfaces. Furthermore, surface texture did not influence Treg frequencies, but we found a significant increase in the proportion of FOXP3+ cells in VelvetSurface® and Polytech texture in comparison to polystyrene. However, a transient activation of Foxp3 may also occur in nonregulatory cells [34] which are suppressive [35]. We cannot rule out that the increase of Foxp3 upon exposure to silicone surface might occur in T cells that mainly have an effector phenotype. In support of our hypothesis is our finding that the increased Foxp3 expression was transient since its levels decreased after 7 days of culture **(S2 Table)**. It has been suggested that only T cells that stably express Foxp3 are regulatory [35].

Using multiplex assay we were able to detect only the proinflammatory cytokines IL-1β, IL-6 and TNF-α in the supernatants of cultured PBMCs. Only the Polytech implant surface showed a significant increase of those cytokines compared to the polystyrene surface. This finding might be explained by the minor increase in the proportion of effector/memory cells. Moreover, as described by Barr et al., the surface texture of Polytech is highly hydrophobic with largest contact angles that reduce the wettability. The roughness may have an influence on the wettability by increasing the hydrophobicity of the surface [16]. These properties allow spreading of macrophages that might produce pro-inflammatory cytokines [16].

Finally, we investigated the effect of each surface on adherent cells at the mRNA level. We found that, among the different surfaces expression levels of CD68 was significantly reduced on SilkSurface. We also observed a downregulation of TNF-α mRNA on SilkSurface®, VelvetSurface® and Micropolyurethane foam surface. IL-10 was downregulated on all surfaces except Polytech foam and Siltex. Moreover, we found a moderate, though not statistically significant, upregulation in TGF-β1 on all surfaces except on the SilkSurface. TGF-β1 has both antiinflammatory and profibrotic properties. It is well known that increased production of TGF-β1 in tissues induces local fibrogenesis and ultimately causes end-stage organ disease [36]. Our findings suggest that certain surface textures are more prone to induce a proinflammatory immune response. Compared to the other surfaces, SilkSurface, VelvetSurface and Micropolyurethane foam surface showed a lower degree of inflammation (downregulation of TNF-α and IL-1β cytokine levels). The fact that TGF-β1 was downregulated only on SilkSurface® suggests that this surface might properly induce a lower degree of fibrosis. However, we do not know how this will be reflected in the *in vivo* situation. Further studies are needed in order to address this issue.

Smooth surface may contain nanoscale roughness and it was shown that macrophages are well spread on this surface compared to the others [4]. In addition we also found a downregulation of monocyte/macrophage markers (CD14, CD68) on certain surfaces with exception of Siltex and polystyrene surfaces. Siltex surface has been reported to have a higher than average level of cell attachment [16, 18]. Furthermore, it is known that macrophages preferentially adhere to polystyrene surface. In agreement with these data, we did not find any down- regulation of CD14 or CD68 or IL-10, which would suggest that monocyte-derived cells are more prone to adhere to these surfaces.

Finally, we have to take into account that in our study we used “naked” silicone surfaces. It was shown that serum coating or coating using other proteins (collagen, fibronectin, etc.) would facilitate the interaction between macrophages/T cells and synthetic biomaterial via the formation of a protein layer that would be rapidly adsorbed on the surface [19]. Moreover, our study investigated the *in vitro* effects of each surface on PBMC in a “static model”, where no mechanical stress occurs in contrast to the dynamic changing occurring during mechanical stress *in vivo*. In fact, it has been shown that the surface of textured implants changes overtime and becomes smoother [37]. In tissue microenvironment, silicone particles might be released from the surface by eliciting T cell/macrophage responses and influencing the cytokine milieu too. Further studies are needed to address this issue.

In conclusion, our *in vitro* study showed that surfaces of commercially available silicone surfaces: a) do not activate T cells, b) do not induce proliferation of T cells, and c) do not alter the distribution of T cell subsets (CD4+, CD8+, naïve, central and effector memory T cells). However, certain surfaces induce mononuclear cells to secrete proinflammatory cytokines that could lead to increased fibrosis.

## Supporting information

S1 Fig Silicone breast implant surface images were acquired with stereomicroscope (scale 200 μm). Details regarding manufacturing, physical characteristic (SEM, roughness, wettability) of those surfaces are described in [16,18,25]. Briefly, all these implants are hydrophobic with the exception of products with smooth surface. Allergan smooth surface shows rocky formations and small pitts and it is made by dipping a mandrel int*o* liquid silicone creating multi layers. SilkSurface® and VelvetSurface® are made without the use of foreign materials (i.e. sugar, salt) and the controlled surface treatment is accomplished through the Motiva 3D Inversion™ Manufacturing Process (source: https://motivaimplants.com/products/). Biocell has a pitted surface with cuboid-shaped wells; it is manufactured using the “salt loss technique”, where salt crystals are added to the silicone mandrel and later washed off. Polytech implants are textured or enveloped in a Micropolyurethane foam. The Micropolyurethane foam surface shows the deepest structure of all textured surfaces. It has a “trabecula” structure building up in layers from its silicone base. Finally, Siltex shows a nodular textured surface and it is made using imprinting manufacture, where the dipped silicone mandrel is pressed into polyurethane foam.
(TIF)

S2 Fig A) Representative dot plots expressing % proliferation. B) CFSE-labeled PBMC were cultured on different silicone surfaces as indicated and stimulated with and without anti-human CD3/CD28 mAbs. Cell proliferation was assessed by FACS after 4 days by CFSE dilution. Each data point represents an individual donor. Results from 2 independent experiments are expressed as mean ± SEM.
(TIF)

S1 Table Percentage of CD4^+^CD25^+^ cells prior plating (day 0) and after 4 days of culture on different silicone surfaces as indicated. Data are shown as mean ± SEM (n = 7).
(DOCX)

S2 Table Percentage of Tregs (CD4^+^CD25^+^Foxp3^+^) after 7 days of culture on different silicone surfaces as indicated. Data are shown as mean ± SEM (n = 7).
(DOCX)
