# Supplementary figures and images for: Immunophenotypic characterization of human T cells after in vitro exposure to different silicone breast implant surfaces
Source: PLoS One. 2018 Feb 8;13(2):e0192108. doi: 10.1371/journal.pone.0192108 (PMC5805229; doi:10.1371/journal.pone.0192108)

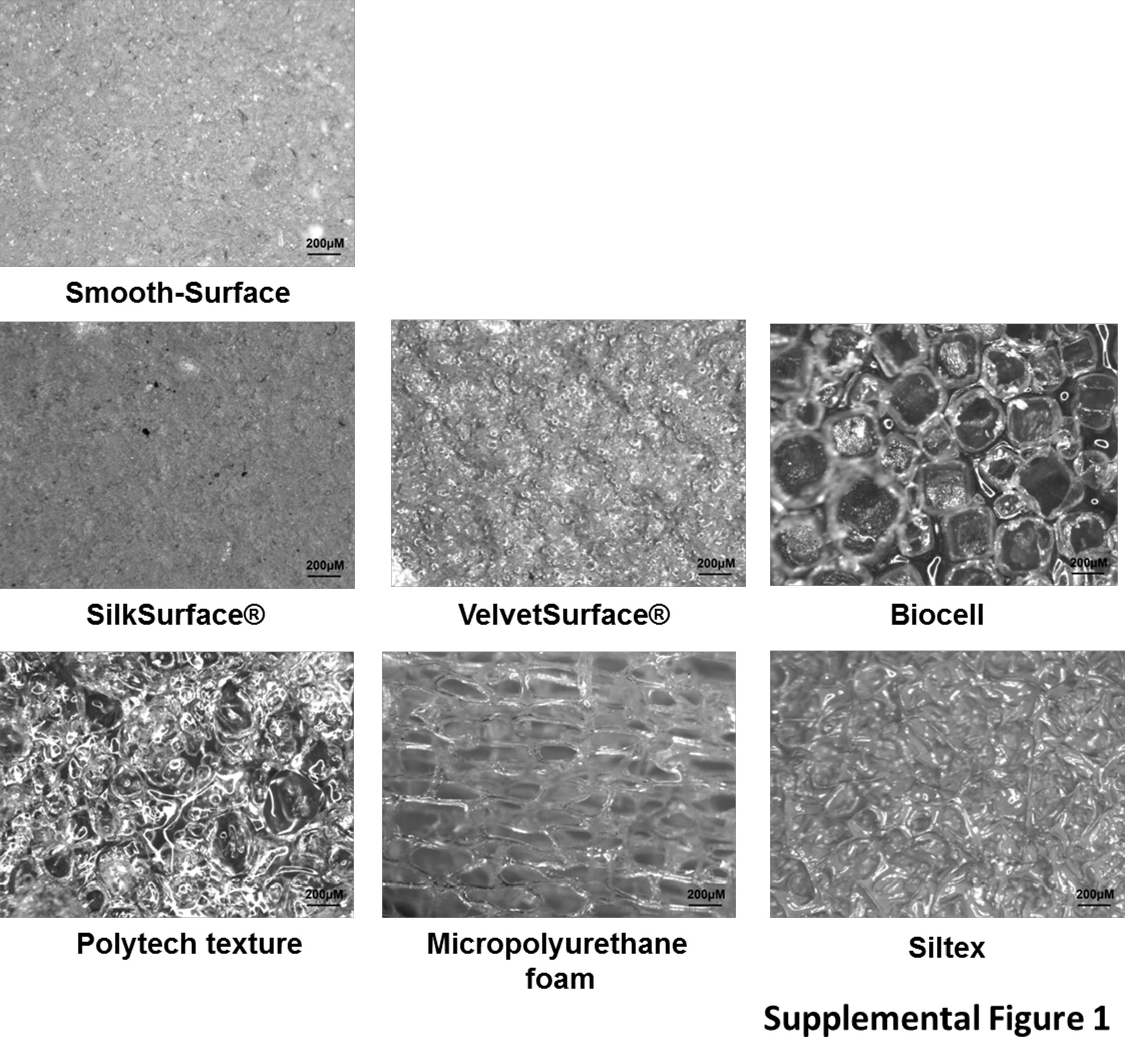

Supplement: S1 Fig — Details regarding manufacturing, physical characteristic (SEM, roughness, wettability) of those surfaces are described in [16,18,25]. Briefly, all these implants are hydrophobic with the exception of products with smooth surface. Allergan smooth surface shows rocky formations and small pitts and it is made by dipping a mandrel into liquid silicone creating multi layers. SilkSurface® and VelvetSurface® are made without the use of foreign materials (i.e. sugar, salt) and the controlled surface treatment is accomplished through the Motiva 3D Inversion™ Manufacturing Process (source: https://motivaimplants.com/products/). Biocell has a pitted surface with cuboid-shaped wells; it is manufactured using the “salt loss technique”, where salt crystals are added to the silicone mandrel and later washed off. Polytech implants are textured or enveloped in a Micropolyurethane foam. The Micropolyurethane foam surface shows the deepest structure of all textured surfaces. It has a “trabecula” structure building up in layers from its silicone base. Finally, Siltex shows a nodular textured surface and it is made using imprinting manufacture, where the dipped silicone mandrel is pressed into polyurethane foam. (TIF) [file pone.0192108.s001.tif]

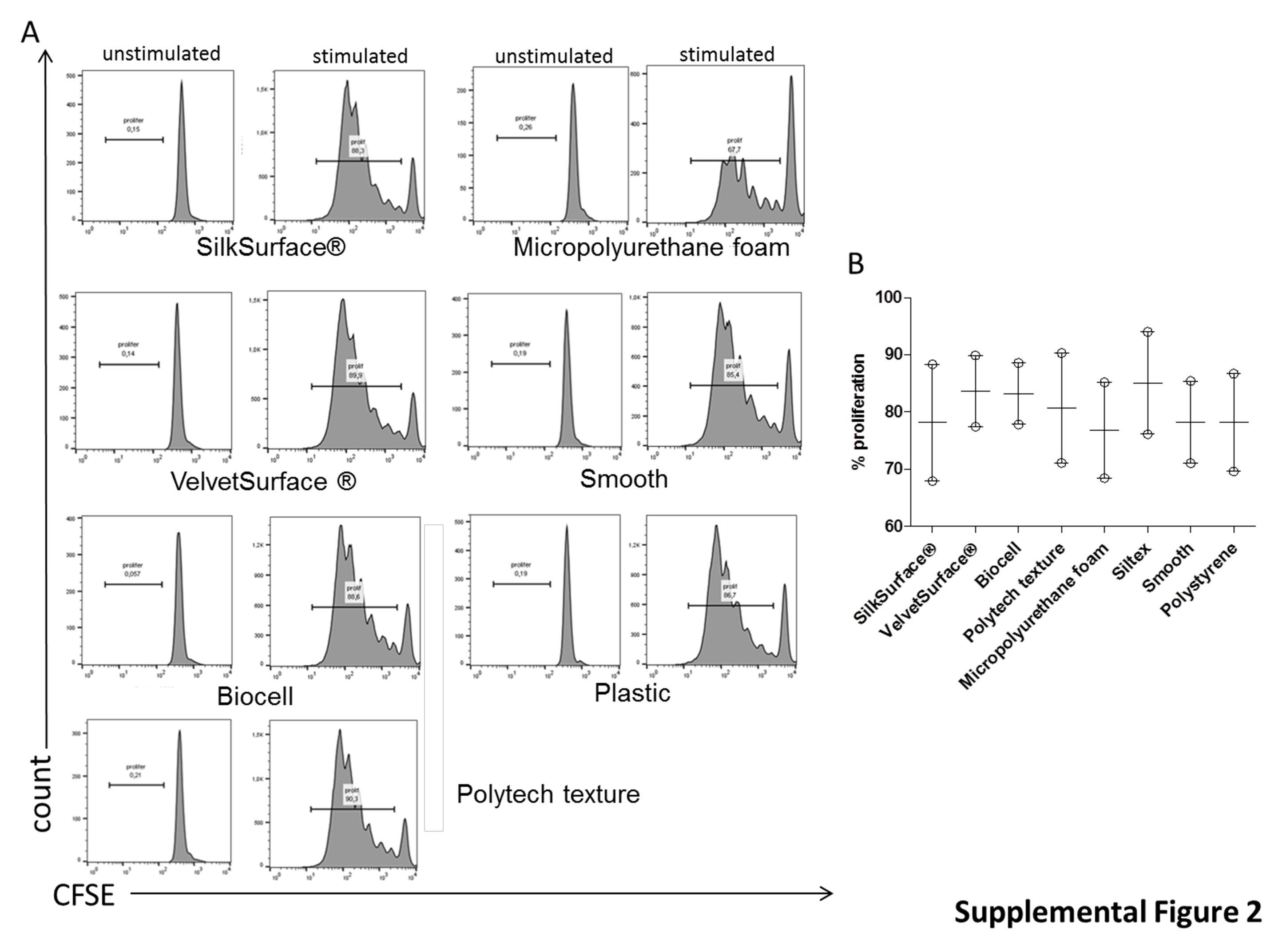

Supplement: S2 Fig — A) Representative dot plots expressing % proliferation. B) CFSE-labeled PBMC were cultured on different silicone surfaces as indicated and stimulated with and without anti-human CD3/CD28 mAbs. Cell proliferation was assessed by FACS after 4 days by CFSE dilution. Each data point represents an individual donor. Results from 2 independent experiments are expressed as mean ± SEM. (TIF) [file pone.0192108.s002.tif]
