# Supplementary material for: Immunophenotypic characterization of human T cells after in vitro exposure to different silicone breast implant surfaces
Source: PLoS One. 2018 Feb 8;13(2):e0192108. doi: 10.1371/journal.pone.0192108 (PMC5805229; doi:10.1371/journal.pone.0192108)
Supplement: S1 Table — Data are shown as mean ± SEM (n = 7). (DOCX) [file pone.0192108.s003.docx]

**S1 Table**

|  |  | **day 4** | | | | | | | |
| --- | --- | --- | --- | --- | --- | --- | --- | --- | --- |
| **%CD4+CD25+** | **day 0** | **SilkSurface®** | **VelvetSurface®** | **Biocell** | **Polytech texture** | **Micropolyurethane foam** | **Siltex** | **Smooth** | **Plastic** |
| mean | 5,6442857 | 5,812857143 | 5,824285714 | 5,702857 | 6,104285714 | 5,475714 | 5,075714 | 5,835714 | 5,545714 |
| SEM | 0,6656727 | 0,600812603 | 0,549859483 | 0,488614 | 0,511529474 | 0,246422 | 0,416839 | 0,414829 | 0,699 |
