# Supplementary material for: Immunophenotypic characterization of human T cells after in vitro exposure to different silicone breast implant surfaces
Source: PLoS One. 2018 Feb 8;13(2):e0192108. doi: 10.1371/journal.pone.0192108 (PMC5805229; doi:10.1371/journal.pone.0192108)
Supplement: S2 Table — Data are shown as mean ± SEM (n = 7). (DOCX) [file pone.0192108.s004.docx]

**S2 Table**

|  | **day 7** | | | | | | | |
| --- | --- | --- | --- | --- | --- | --- | --- | --- |
| **%CD4+CD25+Foxp3+** | **SilkSurface®** | **VelvetSurface®** | **Biocell** | **Polytech texture** | **Micropolyurethane foam** | **Siltex** | **Smooth** | **Plastic** |
| mean | 1,075714 | 1,742857 | 1,022857 | 1,602857 | 1,357143 | 1,49 | 1,501429 | 0,625714 |
| SEM | 0,177867 | 0,336352 | 0,174964 | 0,274394 | 0,126346 | 0,315313 | 0,353725 | 0,076194 |
